# Ondansetron use is associated with increased risk of acute kidney injury in ICU patients following cardiac surgery: a retrospective cohort study

**DOI:** 10.3389/fphar.2024.1511545

**Published:** 2024-12-16

**Authors:** Feiyi Xu, Xun Gong, Wei Chen, Xiaomin Dong, Jiang Li

**Affiliations:** ^1^ Department of Pharmacy, Affiliated Hospital of Guilin Medical University, Guilin, Guangxi, China; ^2^ University of Pharmacy, Guilin Medical University, Guilin, Guangxi, China

**Keywords:** Ondansetron, acute kidney injury, cardiac surgery, 28-day mortality, postoperative atrial fibrillation, MIMIC-IV

## Abstract

**Background:**

Ondansetron is widely used for prophylaxis of postoperative nausea and vomiting (PONV) after general anesthesia. While previous studies have emphasized its early use, the effects of ondansetron in intensive care unit (ICU) patients following cardiac surgery remain unclear. This study investigates the association between postoperative ondansetron exposure and the risk of mortality, acute kidney injury (AKI), and postoperative atrial fibrillation (POAF) in ICU patients after cardiac surgery.

**Methods:**

We conducted a retrospective cohort study utilizing data from the MIMIC-IV database. Adult patients who underwent cardiac surgery and were subsequently admitted to the ICU were included. Cox proportional hazards models were employed to assess the effect of ondansetron exposure on ICU and 28-day mortality. Multivariable logistic regression analyses examined the associations between ondansetron exposure and the incidence of AKI (2-day and 7-day) and POAF. Sensitivity analyses included Propensity Score Matching (PSM) and the inclusion of patients with ICU stays of less than 24 h to ensure robustness of results. Subgroup analyses explored the effects of age and other clinical factors, with interaction tests to examine differential impacts.

**Results:**

A total of 7,170 were included. The incidence of AKI was 74.4% at 2 days and 76.7% at 7 days post-surgery. The 28-day postoperative mortality rate was 1.4%, while the ICU mortality rate was 1.0%. POAF occurred in 17.4% of the patients. Ondansetron exposure was not associated with 28-day mortality or ICU mortality (*p* > 0.05). However, after PSM, ondansetron exposure was significantly associated with an elevated risk of AKI at 2 days (OR 1.28, 95% CI 1.13–1.45, *p* < 0.001] and 7 days (OR 1.25, 95% CI 1.15–1.45, *p* < 0.001), as well as POAF (OR 1.20, 95% CI 1.04–1.39, *p* = 0.014).Subgroup analysis revealed a stronger association in patients aged over 65 years, where ondansetron was linked to an increased risk of 7-day AKI (OR 1.51, 95% CI 1.29–1.78, *p* < 0.001) and POAF (OR 1.31, 95% CI 1.12–1.53, *p* = 0.001). Interaction tests showed a significant interaction between ondansetron exposure and age (P for interaction = 0.018 for AKI and P for interaction = 0.02 for POAF). Sensitivity analyses, including patients with ICU stays of less than 24 h, confirmed the robustness of these findings.

**Conclusion:**

In ICU patients following cardiac surgery, postoperative use of ondansetron is associated with an increased risk of both 7-day AKI and POAF, particularly in patients aged 65 years and older. These findings suggest that the use of ondansetron in this population should be approached with caution, especially in elderly patients who may be more susceptible to these complications. Further research is needed to explore the mechanisms underlying the association between ondansetron and these adverse outcomes.

## 1 Introduction

Postoperative nausea and vomiting (PONV) are prevalent and distressing complications that can significantly impede recovery after surgery. The incidence of PONV is notably high among cardiac surgery patients due to factors such as prolonged operative times, the extensive use of opioids, and the complexity of surgical procedures (Gan et al., 2002). Ondansetron, a selective 5-hydroxytryptamine 3 (5-HT3) receptor antagonist, has been the cornerstone of prophylactic antiemetic therapy to mitigate PONV in this setting (Gan et al., 2020). Its widespread use is attributed to its efficacy and favorable safety profile in the general surgical population.

However, the safety of ondansetron in critically ill patients, particularly those in the intensive care unit (ICU) following cardiac surgery, has come under scrutiny. Acute kidney injury (AKI) is a common and severe complication after cardiac surgery, with incidence rates ranging from 15% to 50% (Brown et al., 2023). AKI in this context is associated with increased morbidity and mortality, prolonged ICU stays, and substantial healthcare costs (Wang and Bellomo, 2017). The etiology of postoperative AKI is multifactorial, involving hemodynamic fluctuations, inflammation, oxidative stress, and nephrotoxic exposures (Hoste and Kellum, 2006).

Recent studies have suggested a potential association between ondansetron use and the risk of AKI in hospitalized patients (Nishtala and Chyou, 2020). While some data indicate that ondansetron may exert nephroprotective effects (Xiong and Xiong, 2022), the evidence is conflicting, and the specific impact on cardiac surgery patients remains unclear. Moreover, ondansetron has been implicated in cardiac arrhythmias, including QT interval prolongation and, in rare cases, torsade’s de pointes (Moeller et al., 2016). Postoperative atrial fibrillation (POAF) is another significant complication after cardiac surgery, affecting up to 40% of patients (Dobrev et al., 2019). POAF is associated with increased risks of stroke, hemodynamic instability, and mortality (William et al., 2024). The potential proarrhythmic effects of ondansetron warrant careful evaluation in this vulnerable population.

Given these concerns, there is an urgent need to re-examine the risk-benefit profile of ondansetron in ICU patients following cardiac surgery. Understanding whether ondansetron contributes to adverse renal and cardiovascular outcomes is crucial for optimizing perioperative care and improving patient safety. This retrospective cohort study aims to investigate the association between postoperative ondansetron exposure and the risks of mortality, AKI, and POAF in cardiac surgery patients admitted to the ICU. Utilizing the extensive data available in the MIMIC-IV database, we seek to provide comprehensive insights that could influence clinical practice guidelines and promote better outcomes in this high-risk group.

## 2 Methods

### 2.1 Database

This study utilized the Medical Information Mart for Intensive Care (MIMIC-IV) electronic database (version 2.2), developed collaboratively by the Massachusetts Institute of Technology (MIT) and Beth Israel Deaconess Medical Center (BIDMC). The database contains relevant information on patients who underwent inpatient treatment at BIDMC between 2008 and 2019. The Institutional Review Board (IRB) of BIDMC waived the requirement for informed consent and allowed the sharing of research resources since all data were de-identified (Johnson et al., 2023). Before data extraction, the author Xiaomin Dong fulfilled all necessary requirements to access the database (Record ID: 57459186).

### 2.2 Study population

**FIGURE 1:**
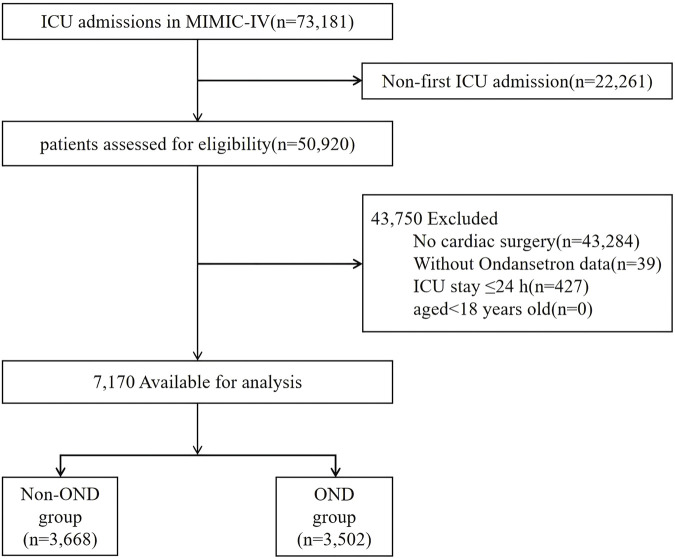
Flowchart of study patients.

Medical records of all adult patients aged 18 years or older who were admitted to the ICU after coronary artery bypass grafting (CABG), valve surgery, or aortic surgery were analyzed. A comprehensive list of cardiac procedure codes is provided in Supplementary Table S1. For patients who underwent more than one cardiac surgery, only the data from their first cardiac procedure admission were included. The exclusion criteria were as follows: (1) patients with multiple ICU admissions for cardiac surgery, for whom only the data from the first admission were considered; (2) patients who did not undergo cardiac surgery; (3) patients with missing data on ondansetron exposure; (4) ICU stay ≤24 h; (5) patients aged under 18 years. After applying these exclusion criteria, a total of 7,170 patients were included in the final analysis (Figure 1).

### 2.3 Data extraction

Data collected included (1) demographic characteristics [gender, age (yr), race]; (2) physiological features and laboratory indicators [mean heart rate, mean blood pressure, mean respiratory rate,mean body temperature, preoperative creatinine, white blood cell (WBC) count, blood urea nitrogen (BUN), chloride, potassium, sodium]; (3) Charlson Comorbidity Index, Acute physiology score III (APS-III), Sequential Organ Failure Assessment (SOFA) score; (4) comorbidities (hypertension, myocardial infarct, sepsis, renal disease, cerebrovascular disease, diabetes, liver disease, chronic pulmonary disease, dementia, peripheral vascular disease, congestive heart failure; (5) treatment measures (mechanical ventilation, antibiotics, vasoactive drugs).

The follow-up period began at hospital admission and ended upon death. All laboratory variables and disease severity scores were obtained from data recorded at the first instance after the patient’s admission to the hospital. Covariates with missing values exceeding 20% were excluded. Covariates with less than 20% missing data were processed using the multiple imputation scheme of the Free Statistics software version 1.9 (Beijing, China) and the statistical software package R 3.3.2.

### 2.4 Clinical outcomes

The primary outcome was postoperative AKI and POAF. AKI was defined by the Kidney Disease: Improving Global Outcomes (KDIGO) guidelines, involving an increase in serum creatinine (SCr) to ≥ 1.5 times the baseline within the prior 7 days; or a rise of ≥0.3 mg/dL in SCr within 48 h; or urine volume of <0.5 mL/kg/h for 6 h or more (Khwaja, 2012). Both serum creatinine levels and urine output measurements were utilized to diagnose AKI, ensuring high sensitivity in detecting cases. The secondary outcomes were 28-day mortality, defined as death within 28 days after surgery, ICU mortality.

### 2.5 Statistical analysis

Continuous variables were reported as median interquartile range (IQR), while categorical variables were expressed as percentages. Fisher’s exact test was used to assess statistical differences between groups for each variable. To evaluate influencing factors related to the risk of AKI and POAF, binary logistic regression analysis was performed.

We prioritized variables that are clinically significant in the context of postoperative cardiac ICU patients. For example, we included factors such as the severity of preoperative disease and the presence of comorbidities, which are critical in assessing patient risk and guiding clinical management in this population. Confounding variables were selected based on a combination of clinical expertise and previous research (Xiong and Xiong, 2022; Cheruku et al., 2023).

In the crude model, covariates were not adjusted. In Model 1, the covariates were adjusted for age, gender, and race. Model 2 and Model 3 were developed based on previous literature and common clinical knowledge to identify characteristic variables that significantly impact the prediction of AKI following cardiac surgery.

Model 2 adjusted for the variables included in Model 1, along with comorbidities such as hypertension, myocardial infarction, renal disease cerebrovascular disease, diabetes, liver disease, chronic pulmonary disease, peripheral vascular disease, and congestive heart failure. Model 3 extended the adjustments of Model 2 by including additional clinical factors such as the use of mechanical ventilation, antibiotics, and vasopressors. It also accounted for the first recorded levels of WBC, chloride, potassium, and sodium. Additionally, physiological parameters such as mean heart rate, mean arterial blood pressure, mean respiratory rate, and mean body temperature, along with the APS III and SOFA score, were included in Model 3.

To mitigate potential confounding, we employed Propensity Score Matching (PSM) to balance baseline characteristics between patients who received ondansetron and those who did not. A 1:1 nearest-neighbor matching algorithm, without replacement, was applied with a caliper width of 0.1. Additionally, a multivariate Cox proportional hazards model was used to assess the association between ondansetron use and mortality.

We performed a subgroup analysis to investigate whether ondansetron had any effect in different subgroups, including age, gender, Charlson comorbidity index, SOFA score, type of surgery, and diabetes. Additionally, we performed a sensitivity analysis by incorporating patients with an ICU stay of less than 24 h following cardiac surgery. For this analysis, we employed logistic regression (Model 3) to evaluate the relationship between ondansetron use and the primary outcomes.

All statistical analyses were performed using R software (version 3.3.2), and *p* < 0.05 was considered statistically significant.

## 3 Results

### 3.1 Population

A total of 7,170 patients who underwent postoperative cardiac surgery were included in this study (Figure 1). The median age of the cohort was 68.7 years (interquartile range [IQR]: 60.9–76.2 years). The incidence of AKI was 74.4% at 2 days and 76.7% at 7 days post-surgery. The 28-day postoperative mortality rate was 1.4%, while the ICU mortality rate was 1.0%. POAF occurred in 17.4% of the patients. Baseline characteristics and clinical outcomes, stratified by ondansetron exposure, are presented in Table 1.

**TABLE 1:** Characteristics of patients by fibrinogen level.

Characteristics	Total	Non-OND group	OND group	P
	(n = 7,170)	(n = 3,668)	(n = 3,502)	
Hospitalization status				
Gender, Male (%)	5,115 (71.3)	2,678 (73.0)	2,437 (69.6)	0.001
Age, years	68.7 (60.9, 76.2)	68.5 (60.9, 76.4)	68.9 (61.0, 76.0)	0.921
Race, White (%)	5,256 (73.3)	2,742 (74.8)	2,514 (71.8)	0.018
MHR, beats/min	81.1 (75.7, 87.6)	82.1 (76.5, 88.6)	80.2 (75.0, 86.4)	<0.001
MBP, mmHg	74.0 (70.3, 78.0)	74.2 (70.4, 78.1)	73.8 (70.2, 78.0)	0.136
MRR, beats/min	17.5 (15.9, 19.3)	17.4 (15.8, 19.1)	17.6 (16.1, 19.4)	<0.001
Temperature, °C	36.7 (36.5, 36.9)	36.7 (36.5, 37.0)	36.7 (36.5, 36.9)	<0.001
Comorbidities				
Hypertension, n (%)	4,039 (56.3)	2,100 (57.3)	1939 (55.4)	0.108
Myocardia Infarct, n (%)	2074 (28.9)	1,025 (27.9)	1,049 (30)	0.061
Sepsis, n (%)	4,102 (57.2)	2,592 (70.7)	1,510 (43.1)	<0.001
Renal Diseases, n (%)	1,184 (16.5)	552 (15)	632 (18)	<0.001
Cerebrovascular Disease, n (%)	746 (10.4)	416 (11.3)	330 (9.4)	0.008
Diabetes, n (%)	2,540 (35.4)	1,277 (34.8)	1,263 (36.1)	0.268
Liver Disease, n (%)	283 (3.9)	154 (4.2)	129 (3.7)	0.263
Chronic Pulmonary Disease, n (%)	1,551 (21.6)	903 (24.6)	648 (18.5)	<0.001
Dementia, n (%)	33 (0.5)	9 (0.2)	24 (0.7)	0.006
Peripheral Vascular Disease, n (%)	1,210 (16.9)	655 (17.9)	555 (15.8)	0.023
Congestive Heart Failure, n (%)	1969 (27.5)	1,069 (29.1)	900 (25.7)	0.001
Scoring systems				
Charlson Comorbidity Index	5.0 (4.0, 6.0)	5.0 (4.0, 6.0)	5.0 (4.0, 6.0)	0.889
APS-III	35.0 (27.0, 47.0)	36.0 (28.0, 48.0)	34.0 (27.0, 45.0)	<0.001
SOFA	5.0 (4.0, 7.0)	5.0 (4.0, 7.0)	5.0 (4.0, 7.0)	<0.001
Laboratory tests				
Creatinine, (mg/dL)	0.9 (0.7,1.1)	0.9 (0.7,1.1)	0.9 (0.7,1.1)	0.178
WBC, (K/µL)	12.2 (9.1, 15.7)	12.0 (9.0, 15.8)	12.3 (9.2, 15.6)	0.397
BUN, (mg/dL)	16.0 (13.0, 21.0)	17.0 (13.0, 22.0)	16.0 (13.0, 20.0)	<0.001
Chloride, (mEq/L)	109.0 (106.0, 111.0)	109.0 (107.0, 112.0)	108.0 (106.0, 110.0)	<0.001
Potassium, (mEq/L)	4.3 (4.0, 4.6)	4.2 (3.9, 4.5)	4.3 (4.0, 4.7)	<0.001
Sodium, (mEq/L)	139.0 (137.0, 141.0)	139.0 (137.0, 141.0)	139.0 (137.0, 140.0)	<0.001
Treatment measures				
Mechvent Ventilation, n (%)	4,579 (63.9)	2,229 (60.8)	2,350 (67.1)	<0.001
Antibiotics, n (%)	6,914 (96.4)	3,494 (95.3)	3,420 (97.7)	<0.001
Vasoactive Drug, n (%)	5,357 (74.7)	2,812 (76.7)	2,545 (72.7)	<0.001

Notes: OND: ondansetron; MHR: mean heart rate; MBP: mean arterial blood pressure; MRR: mean respiratory rate; WBC: white blood cell; BUN: blood urea nitrogen; APSIII: Acute physiology score III; SOFA: sequential organ failure assessment.

Analysis revealed that patients who received ondansetron postoperatively were more likely to be female and had lower APS III scores. These patients also exhibited fewer comorbidities, including congestive heart failure, peripheral vascular disease, chronic lung disease, and sepsis. Additionally, they had a lower incidence of requiring vasoactive drugs. However, they had a higher prevalence of chronic renal disease and were more likely to need mechanical ventilation (Table 1).

The 1:1 propensity score matching algorithm applied to the dataset of patients receiving ondansetron resulted in 5,204 matched pairs. In the propensity-matched cohorts, the standardized mean differences for all baseline characteristics were below 0.10, indicating minimal imbalance between the groups (Supplementary Table S2).

### 3.2 2-Day AKI

Logistic regression analysis was conducted to evaluate the relationship between ondansetron use and 2-day AKI. Ondansetron was identified as a significant risk factor in all models. In the unadjusted model, the odds ratio (OR) was 1.41 (95% CI: 1.26–1.57; *p* < 0.001). After adjusting for multiple covariates in Model 3, the OR remained significant at 1.36 (95% CI: 1.21–1.52; *p* < 0.001) (Table 2). These findings were further corroborated in the propensity-matched cohort, where ondansetron exposure was associated with an OR of 1.28 (95% CI: 1.13–1.45; *p* < 0.001), reinforcing the robustness of the association between ondansetron use and increased risk of 2-day AKI (Table 3).

**TABLE 2:** Relationship between Ondansetron and outcomes (Logistic).

Quartiles		OR (95% CI)									
		No. total	No. event%	Unadjusted	P	Model1	P	Model2	P	Model3	P
2-day AKI	Non-OND users	3,668	2,613 (71.2)	1(Ref)		1(Ref)		1(Ref)		1(Ref)	
	OND users	3,502	2,721 (77.7)	1.41 (1.26–1.57)	<0.001	1.42 (1.27–1.58)	<0.001	1.43 (1.28–1.6)	<0.001	1.36 (1.21–1.52)	<0.001
7-day AKI	Non-OND users	3,668	2,716 (74.0)	1(Ref)		1(Ref)		1(Ref)		1(Ref)	
	OND users	3,502	2,783 (79.5)	1.36 (1.21–1.51)	<0.001	1.36 (1.22–1.52)	<0.001	1.38 (1.24–1.55)	<0.001	1.32 (1.17–1.49)	<0.001
POAF	Non-OND users	3,668	612 (16.7)	1(Ref)		1(Ref)		1(Ref)		1(Ref)	
	OND users	3,502	638 (18.2)	1.11 (0.98–1.26)	0.087	1.12 (0.99–1.27)	0.064	1.17 (1.03–1.33)	0.014	1.19 (1.04–1.37)	0.01

Model 1 adjusted for age, race and gender.

Model 2 adjusted for model 1, hypertensive, myocardial infarct, congestive heart failure, peripheral vascular disease, cerebrovascular disease, renal disease, diabetes, liver disease, and chronic pulmonary disease.

Model 3 adjusts for model 2, mechanical ventilation, use of vasopressors, use of antibiotics, first sodium, first potassium, first chloride, first white blood cell, along with mean heart rate, mean arterial blood pressure, mean respiratory rate, mean temperature, Acute Physiology Score III (APS III), and Sequential Organ Failure Assessment (SOFA).

**TABLE 3:** Association between ondansetron and 2-day AKI and 7-day AKI in patients with Cardiac Surgery.

	Models	OR (95%CI)	P
2-day AKI	Propensity Score. adjusted	1.32 (1.18–1.48)	<0.001
	Propensity Score. Matched	1.28 (1.13–1.45)	<0.001
7-day AKI	Propensity Score. adjusted	1.29 (1.15–1.45)	<0.001
	Propensity Score. Matched	1.25 (1.10–1.42)	0.001
POAF	Propensity Score. adjusted	1.17 (1.03–1.33)	0.014
	Propensity Score. Matched	1.20 (1.04–1.39)	0.014

### 3.3 7-Day AKI

Similarly, logistic regression analysis for 7-day AKI revealed that ondansetron use was a significant risk factor in all models. In the unadjusted model, the OR was 1.36 (95% CI: 1.21–1.51; *p* < 0.001). In Model 3, after adjusting for multiple covariates, the OR was 1.32 (95% CI: 1.17–1.49; *p* < 0.001) (Table 2). Results from the propensity-matched cohort showed an OR of 1.25 (95% CI: 1.10–1.42; *p* < 0.001), further supporting the strong association between ondansetron exposure and an increased risk of 7-day AKI (Table 3).

### 3.4 POAF

Logistic regression analysis was also employed to examine the relationship between ondansetron use and POAF. There was no significant difference in POAF risk between patients exposed and unexposed to ondansetron in the unadjusted model (OR 1.11, 95% CI: 0.98–1.26, *p* = 0.087). However, in Model 3, after adjusting for multiple covariates, the OR was 1.19 (95% CI: 1.04–1.37, *p* = 0.01) (Table 2). Additionally, results from the propensity-matched cohort revealed an OR of 1.20 (95% CI: 1.04–1.39; *p* = 0.014), confirming the robustness of the association between ondansetron exposure and increased POAF risk (Table 3).

### 3.5 ICU mortality

Cox proportional hazards analysis was used to assess the relationship between ondansetron and ICU mortality. No significant difference in ICU mortality risk was found between the ondansetron-exposed and unexposed groups in any of the models. In the unadjusted model, the OR was 1.24 (95% CI: 0.78–1.97; *p* = 0.370), and in Model 1, the OR was 1.19 (95% CI: 0.75–1.89; *p* = 0.467) (Table 4).

**TABLE 4:** Relationship between Ondansetron and outcomes (Cox).

Quartiles		HR (95% CI)					
		No. total	No. event%	Crude	P	Model1	P
ICU mortality	Non-OND users	3,668	33 (0.9)	1(Ref)		1(Ref)	
	OND users	3,502	39 (1.1)	1.24 (0.78–1.97)	0.370	1.19 (0.75–1.89)	0.467
28-day mortality	Non-OND users	3,668	54 (1.5)	1(Ref)		1(Ref)	
	OND users	3,502	47 (1.3)	0.91 (0.62–1.35)	0.638	0.88 (0.60–1.31)	0.539

Model 1 adjusted for age, race, and gender.

### 3.6 28-Day mortality

Similarly, no significant difference in 28-day mortality risk was found between the groups exposed and unexposed to ondansetron. In the unadjusted model, the OR was 0.91 (95% CI: 0.62–1.35; *p* = 0.638), and in Model 1, the OR was 0.88 (95% CI: 0.60–1.31; *p* = 0.539) (Table 4).

### 3.7 Subgroup analysis

**FIGURE 2:**
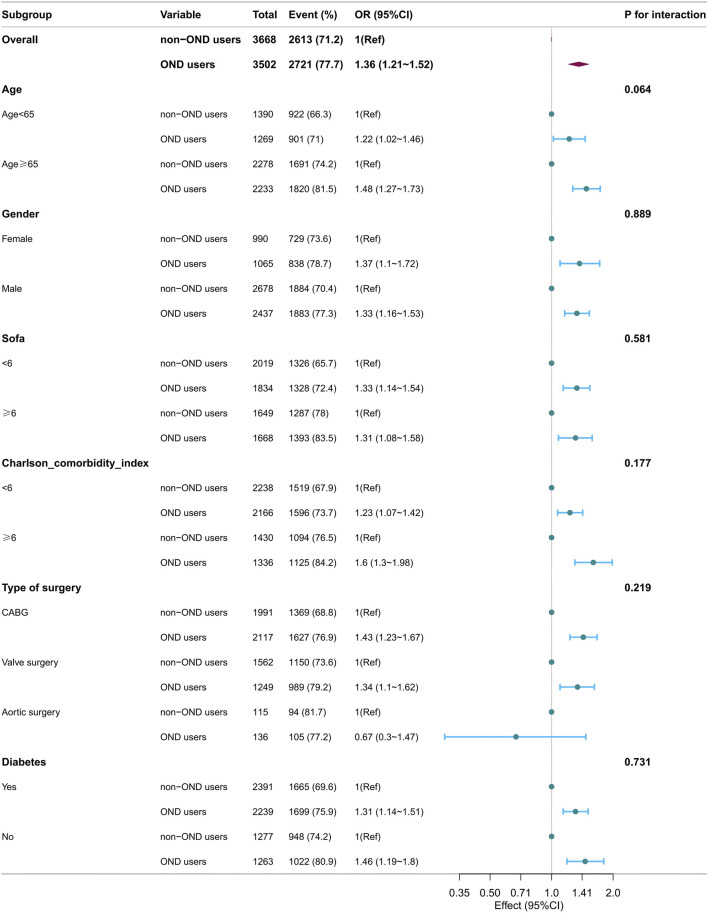
Forest plots of hazard ratios for the 2-day AKI in different subgroups.

In the subgroup analysis for 2-day AKI (Figure 2), no significant interaction was observed between ondansetron exposure and any of the variables examined, including gender (female vs. male), age groups (≥65 years vs. <65 years), Charlson comorbidity index categories (<6 vs. ≥6), SOFA scores (<6 vs. ≥6), types of surgery (coronary artery bypass grafting [CABG], valve surgery, and aortic surgery), and diabetes status (all P for interaction >0.05).

**FIGURE 3:**
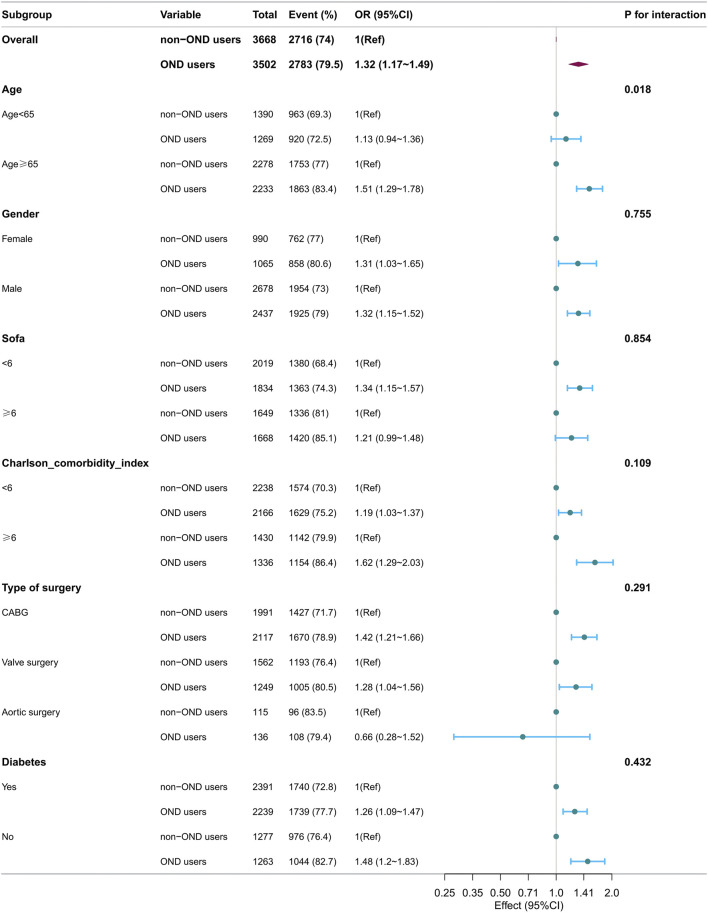
Forest plots of hazard ratios for the 7-day AKI in different subgroups.

**FIGURE 4:**
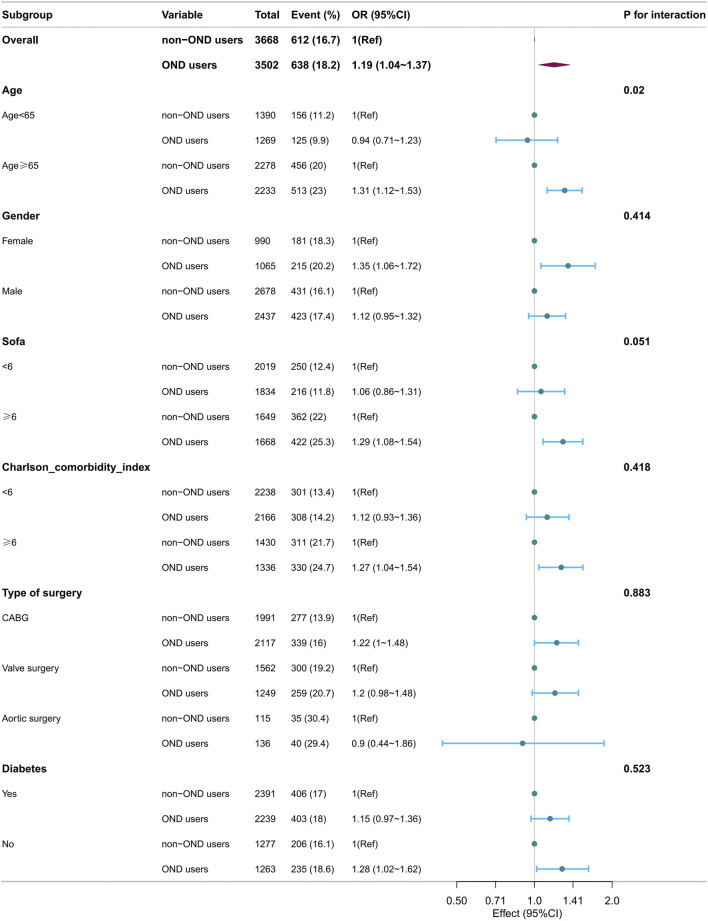
Forest plots of hazard ratios for the POAF in different subgroups.

However, stratified analysis for 7-day AKI (Figure 3) revealed significant interactions between ondansetron exposure and age groups. The association between ondansetron and the increased risk of 7-day AKI was notably stronger in patients aged ≥65 years (OR 1.51, 95% CI: 1.29–1.78; *p* < 0.001) compared to those younger than 65 years (OR 1.13, 95% CI: 0.94–1.36; *p* = 0.178). The interaction between age and ondansetron exposure was significant (P for interaction = 0.018). Similarly, the association between ondansetron and increased POAF risk was stronger in patients aged ≥65 years (OR 1.31, 95% CI: 1.12–1.53; *p* = 0.001) compared to those younger than 65 years (OR 0.94, 95% CI: 0.71–1.23; *p* = 0.653) (Figure 4), with a significant interaction observed (P for interaction = 0.020). No significant interactions were observed in other subgroups.

### 3.8 Sensitivity analysis

The final cohort included 7,170 patients, with 427 additional patients whose ICU stay was less than 24 h included in the analysis, bringing the total to 7,597 patients. Sensitivity analysis indicated that ondansetron use was associated with a higher risk of both 2-day AKI [OR 1.45 (95% CI: 1.30–1.62); *p* < 0.001] and 7-day AKI [OR 1.43 (95% CI: 1.28–1.61); *p* < 0.001]. Furthermore, ondansetron exposure was associated with a higher risk of POAF [OR 1.22 (95% CI: 1.06–1.39); *p* = 0.004] (Table 5).

**TABLE 5:** Sensitivity analysis of the relationship between ICU stay <24 h and primary outcomes.

Quartiles		HR (95% CI)					
		No. total	No. event%	Crude	P	Model3	P
2-day AKI	Non-OND users	3,958	2,698 (68.2)	1(Ref)		1(Ref)	
	OND users	3,639	2,784 (76.5)	1.52 (1.37–1.68)	<0.001	1.45 (1.30–1.62)	<0.001
7-day AKI	Non-OND users	3,958	2,801 (70.8)	1(Ref)		1(Ref)	
	OND users	3,639	2,846 (78.2)	1.48 (1.34–1.65)	<0.001	1.43 (1.28–1.61)	<0.001
POAF	Non-OND users	3,958	619 (15.6)	1(Ref)		1(Ref)	
	OND users	3,639	638 (17.5)	1.15 (1.02–1.29)	0.027	1.22 (1.06–1.39)	0.004

Model 3 adjusts for model 2, mechanical ventilation, use of vasopressors, use of antibiotics, first sodium, first potassium, first chloride, first white blood cell, along with mean heart rate, mean arterial blood pressure, mean respiratory rate, mean temperature, Acute Physiology Score III (APS III), and Sequential Organ Failure Assessment (SOFA).

## 4 Discussion

Our study demonstrates that postoperative use of ondansetron in patients undergoing cardiac surgery is associated with an increased risk of both 2-day and 7-day AKI, while no significant increase in 28-day mortality or ICU mortality was observed. Importantly, ondansetron exposure was also linked to a higher incidence of postoperative atrial fibrillation (POAF). Notably, the association between ondansetron and both 7-day AKI and POAF was particularly pronounced in patients aged 65 years and older, suggesting that age may be an important modifier of the drug’s effects.

Our finding that ondansetron use increases the risk of AKI is consistent with a case-crossover study using a nationally representative sample from New Zealand (Nishtala and Chyou, 2020). However, a contrasting study reported that ondansetron exposure following cardiac surgery was associated with a reduced risk of 7-day AKI (Xiong and Xiong, 2022), highlighting the discrepancies in the existing literature. These differences may stem from variations in study populations and methodologies. Our study included all patients who received ondansetron after cardiac surgery in the MIMIC-IV database, whereas the other study focused on patients who received ondansetron within 48 h following coronary artery bypass grafting (CABG) and valve surgery in the MIMIC-III database. Another notable discrepancy concerns the reported incidence of 7-day AKI (AKI-7d). The earlier study reported an AKI-7d incidence of 45.5%, whereas our study found a significantly higher incidence of 79.6%. This variation is likely due to differences in sample sizes, with 721 cases in the ondansetron group in the previous study, compared to 3,502 cases in our cohort. Additionally, the previous study relied solely on serum creatinine levels to diagnose AKI, while our study utilized both serum creatinine and urine output criteria. This dual approach likely enhanced the sensitivity of AKI diagnosis in our study, contributing to the observed difference in incidence rates.

Physiological studies have confirmed that during cardiopulmonary bypass in cardiac surgery, plasma concentrations of catecholamines such as adrenaline and noradrenaline peak (Levey et al., 2009). Excessively high levels of these endogenous hormones can lead to hemodynamic instability and systemic vasoconstriction, resulting in decreased renal perfusion and ultimately leading to AKI (Schrier and Abraham, 1999). Additionally, previous research has demonstrated that ondansetron can increase the secretion of vasopressin, which may contribute to renal vasoconstriction and reduced glomerular filtration rate, providing a possible mechanism by which ondansetron induces AKI (Barreca et al., 1996). These findings underscore the need for further research to confirm the association between ondansetron and AKI and to elucidate the underlying mechanisms.

Our study found that ondansetron exposure after cardiac surgery was not associated with increased patient mortality, a result that contrasts with some previous retrospective studies. For instance, a recent study reported that ondansetron use was linked to a significant reduction in the risk of in-hospital and 60-day mortality by 38% and 31%, respectively, after propensity score matching and multivariate regression analysis (Tao et al., 2023). Another retrospective cohort study demonstrated that daily low-dose and medium-dose ondansetron improved in-hospital mortality in ICU patients with cardiovascular disease, with odds ratios of 0.36 and 0.26, respectively (Fang et al., 2022). Several factors may explain these inconsistent results. First, the study populations differed: our study specifically focused on ICU patients after cardiac surgery, while the other studies included mechanically ventilated patients and a broader ICU population. Second, the study endpoints varied; we assessed 28-day mortality and ICU mortality in ondansetron users at any time, while the other studies examined 60-day mortality and in-hospital mortality in early ondansetron users. These differences underscore the importance of context when interpreting the effects of ondansetron and suggest that patients undergoing cardiac surgery may require more cautious use of this medication.

Regarding POAF, although case reports have suggested a potential link between ondansetron and new-onset atrial fibrillation, higher-level evidence remains lacking (Havrilla et al., 2009; Kasinath et al., 2003). Our study provides stronger evidence indicating that ondansetron exposure is associated with an increased risk of POAF in ICU patients following cardiac surgery. This finding underscores the need for careful consideration of ondansetron use in this population. Prolonged QT interval, commonly observed in patients receiving ondansetron (Trivedi et al., 2016), has been linked to an increased risk of arrhythmias, and our results suggest that ondansetron exposure may exacerbate this risk. A potential mechanism for the development of POAF could be that prolonged QTc intervals impair ventricular diastolic function, leading to increased atrial wall tension and alterations in atrial substrate, which may trigger atrial fibrillation (Zhang et al., 2018). Further studies are warranted to confirm the safety profile of ondansetron in the cardiac surgery population, given the potential implications for post-operative arrhythmias and overall patient outcomes.

The results of the subgroup analysis are also noteworthy. We found that the association between ondansetron exposure and the increased risk of both 7-day AKI and post-operative atrial fibrillation (POAF) was more pronounced in patients aged 65 years and older. Several factors may contribute to this observation. The elderly are more vulnerable to declines in renal function due to the natural aging process (Fang et al., 2020) and may have higher levels of vasopressin (Plasencia et al., 2019), which could exacerbate the vasoconstrictive effects of ondansetron. Moreover, long-term ondansetron use may have a more significant impact on both renal function and the risk of arrhythmias in this age group (Barreca et al., 1996). Additionally, age-related changes in myocardial and renal function may heighten the susceptibility to these adverse outcomes. These findings suggest that elderly patients may require more cautious use of ondansetron after cardiac surgery, given their increased risk of both renal dysfunction and arrhythmias.

## 5 Limitations

This study has several limitations. First, as a retrospective observational study, it is inherently susceptible to biases and confounding factors that cannot be completely controlled. Second, due to limitations of the MIMIC-IV database, we were unable to account for the dosage of ondansetron, which may introduce potential bias into the results. Additionally, other unmeasured variables, such as intraoperative factors and fluid management, could have influenced the outcomes. Therefore, prospective studies with detailed dosage information and standardized protocols are necessary to validate our findings and to explore the causal relationships further.

## 6 Conclusion

In ICU patients following cardiac surgery, postoperative use of ondansetron is associated with an increased risk of both 7-day AKI and POAF, particularly in patients aged 65 years and older. These findings suggest that the use of ondansetron in this population should be approached with caution, especially in elderly patients who may be more susceptible to these complications. Further research is needed to explore the mechanisms underlying the association between ondansetron and these adverse outcomes.

## Data Availability

The original contributions presented in the study are included in the article/Supplementary Material, further inquiries can be directed to the corresponding authors.

## References

[B1] Barreca T. Corsini G. Cataldi A. Garibaldi A. Cianciosi P. Rolandi E. (1996). Effect of the 5-HT3 receptor antagonist ondansetron on plasma AVP secretion: a study in cancer patients. Biomed. Pharmacother. 50, 512–514. 10.1016/s0753-3322(97)89285-2 9091068

[B2] Brown J. R. Baker R. A. Shore-Lesserson L. Fox A. A. Mongero L. B. Lobdell K. W. (2023). The society of thoracic surgeons/society of cardiovascular anesthesiologists/American society for extracorporeal Technology clinical practice guidelines for the prevention of adult cardiac surgery-associated acute kidney injury. Anesth. Analg. 136, 176–184. 10.1213/ANE.0000000000006286 36534719

[B3] Cheruku S. R. Raphael J. Neyra J. A. Fox A. A. (2023). Acute kidney injury after cardiac surgery: prediction, prevention, and management. Anesthesiology 139 (6), 880–898. 10.1097/ALN.0000000000004734 37812758 PMC10841304

[B4] Dobrev D. Aguilar M. Heijman J. Guichard J. B. Nattel S. (2019). Postoperative atrial fibrillation: mechanisms, manifestations and management. Nat. Rev. Cardiol. 16 (7), 417–436. 10.1038/s41569-019-0166-5 30792496

[B5] Fang Y. Gong A. Y. Haller S. T. Dworkin L. D. Liu Z. Gong R. (2020). The ageing kidney: molecular mechanisms and clinical implications. Ageing Res. Rev. 63, 101151. 10.1016/j.arr.2020.101151 32835891 PMC7595250

[B6] Fang Y. Xiong C. Wang X. (2022). Association between early ondansetron administration and in-hospital mortality in critically ill patients: analysis of the MIMIC-IV database. J. Transl. Med. 20 (1), 223. 10.1186/s12967-022-03401-y 35568908 PMC9107069

[B7] Gan T. J. Belani K. G. Bergese S. Chung F. Diemunsch P. Habib A. S. (2020). Fourth consensus guidelines for the management of postoperative nausea and vomiting. Anesth. Analg. 131, 411–448. 10.1213/ane.0000000000004833 32467512

[B8] Gan T. J. (2002). Postoperative nausea and vomiting—can it be eliminated? JAMA 287 (10), 1233–1236. 10.1001/jama.287.10.1233 11886298

[B9] Havrilla P. L. Kane-Gill S. L. Verrico M. M. Seybert A. L. Reis S. E. (2009). Coronary vasospasm and atrial fibrillation associated with ondansetron therapy. Ann. Pharmacother. 43 (3), 532–536. 10.1345/aph.1L544 19261954

[B10] Hoste E. A. Kellum J. A. (2006). Acute kidney injury: epidemiology and diagnostic criteria. Curr. Opin. Crit. Care 12 (6), 531–537. 10.1097/MCC.0b013e3280102af7 17077682

[B11] Johnson A. Bulgarelli L. Shen L. Gayles A. Shammout A. Horng S. (2023). MIMIC-IV, a freely accessible electronic health record dataset. Sci. DATA 10 (1), 1. 10.1038/s41597-022-01899-x 36596836 PMC9810617

[B12] Kasinath N. S. Malak O. Tetzlaff J. (2003). Atrial fibrillation after ondansetron for the prevention and treatment of postoperative nausea and vomiting: a case report. Can. J. Anaesth. 50, 229–231. 10.1007/BF03017789 12620943

[B13] Khwaja A. (2012). KDIGO clinical practice guidelines for acute kidney injury. Nephron. Clin. Pract. 120 (4), c179–c184. 10.1159/000339789 22890468

[B14] Levey A. S. Stevens L. A. Schmid C. H. Zhang Y. L. Castro A. R. Feldman H. I. (2009). A new equation to estimate glomerular filtration rate. Ann. Intern Med. 150 (9), 604–612. 10.7326/0003-4819-150-9-200905050-00006 19414839 PMC2763564

[B15] Moeller J. R. Gummin D. D. Nelson T. J. Drendel A. L. Shah B. K. Berger S. (2016). Risk of ventricular arrhythmias and association with ondansetron. J. Pediatr. 179, 118–123. 10.1016/j.jpeds.2016.08.058 27665040

[B16] Nishtala P. S. Chyou T. Y. (2020). Identifying drug combinations associated with acute kidney injury using association rules method. Pharmacoepidemiol Drug Saf. 29 (4), 467–473. 10.1002/pds.4960 32080933

[B17] Plasencia G. Luedicke J. M. Nazarloo H. P. Carter C. S. Ebner N. C. (2019). Plasma oxytocin and vasopressin levels in young and older men and women: functional relationships with attachment and cognition. Psychoneuroendocrinology 110, 104419. 10.1016/j.psyneuen.2019.104419 31606581 PMC6943921

[B18] Schrier R. W. Abraham W. T. (1999). Hormones and hemodynamics in heart failure. N. Engl. J. Med. 341 (8), 577–585. 10.1056/NEJM199908193410806 10451464

[B19] Tao L. Chen Y. Chang P. An S. (2023). Association between ondansetron use and mortality of patients on mechanical ventilation in the intensive care unit: a retrospective cohort study. Ann. Transl. Med. 11 (2), 43. 10.21037/atm-22-6256 36819561 PMC9929838

[B20] Trivedi S. Schiltz B. Kanipakam R. Bos J. M. Ackerman M. J. Ouellette Y. (2016). Effect of ondansetron on QT interval in patients cared for in the PICU. Pediatr. Crit. Care Med. 17 (7), e317–e323. 10.1097/PCC.0000000000000776 27387786

[B21] Wang Y. Bellomo R. (2017). Cardiac surgery-associated acute kidney injury: risk factors, pathophysiology and treatment. Nat. Rev. Nephrol. 13 (11), 697–711. 10.1038/nrneph.2017.119 28869251

[B22] William J. Rowe K. Hogarty J. Xiao X. Shirwaiker A. Bloom J. E. (2024). Predictors of late atrial fibrillation recurrence after cardiac surgery. JACC Clin. Electrophysiol. 10 (7 Pt 2), 1711–1719. 10.1016/j.jacep.2024.05.030 39084745

[B23] Xiong D. Xiong C. (2022). Early postoperative ondansetron exposure is associated with reduced 90-day mortality in patients undergoing cardiac surgery. Front. Surg. 9, 885137. 10.3389/fsurg.2022.885137 35784927 PMC9243460

[B24] Zhang N. Gong M. Tse G. Zhang Z. Meng L. Yan B. P. (2018). Prolonged corrected QT interval in predicting atrial fibrillation: a systematic review and meta-analysis. Pacing Clin. Electrophysiol. 41 (3), 321–327. 10.1111/pace.13292 29380395

