# Supplementary material for: Ondansetron use is associated with increased risk of acute kidney injury in ICU patients following cardiac surgery: a retrospective cohort study
Source: Front Pharmacol. 2024 Dec 16;15:1511545. doi: 10.3389/fphar.2024.1511545 (PMC11683680; doi:10.3389/fphar.2024.1511545)
Supplement: Supplementary file 1 [file Table1.docx]

**Supplementary Table 1. All cardiac procedure codes.**

| **Cardiac procedure codes** |
| --- |

| ICD9/ICD10 | 3615, 3522, 3615, 3521, 021109W, 3521, 3612, 3512, 3614, 3614, 02RF08Z, 3845, 02100A9, 3522, 02100Z9, 02100Z8, 021009W, 3612, 3613, 3613, 02RX0JZ, 02110AW, 3523, 021309W, 3611, 3523, 212093, 3611, 02RG08Z, 3524, 3845, 021209W, 02100AW, 02RF0JZ, 3511, 02RW0JZ, 210099, 02110Z9, 02120Z9, 3524, 02RG0JZ, 02100A8, 3514, 213099, 0210099, 02RJ08Z, 02RF0KZ, 3511, 02QG0ZZ, 0212093, 3616, 02110Z8, 02120AW, 3527, 213093, 3527, 02RG0KZ, 02RX08Z, 02100A3, 0213099, 3514, 212099, 210093, 02QF0ZZ, 3616, 211093, 0211093, 02110Z3, 0212099, 0210093, 02110A9, 211099, 0211099. |
| --- | --- |

**Supplementary Table 2. Baseline characteristics of ICU patients by propensity-matched ICU patients ondansetron vs Non-ondansetron.**

|  | **Before PSM** | | | **After PSM** | | |
| --- | --- | --- | --- | --- | --- | --- |
| **Characteristic** | **Non-OND**  **group**  **(n =** 3,668) | **OND**  **group**  **(n = 3,502)** | **SMD** | **Non-OND**  **group**  **(n =** 2,602) | **OND**  **group**  **(n =** 2,602) | **SMD** |
| **Hospitalization status** | | | | | | |
| Gender, Male(%) | 2,678.0(73.0) | 2,437.0(69.6) | 0.08 | 1,955.0(72.0) | 1,922.0(70.8) | 0.03 |
| Age, years | 68.5 (60.9, 76.4) | 68.9 (61.0, 76.0) | 0 | 68.4 (60.9, 76.4) | 68.9 (60.9, 76.1) | -0.01 |
| Race, White (%) | 2,742.0(74.8) | 2,514.0(71.8) | -0.06 | 1,989.0(73.3) | 1,985.0(73.1) | -0.01 |
| MHR, beats/min | 82.1 (76.5, 88.6) | 80.2 (75.0, 86.4) | 0.16 | 81.2 (75.8, 87.5) | 81.0 (75.7, 87.5) | -0.01 |
| MBP, mmHg | 74.2 (70.4, 78.1) | 73.8 (70.2, 78.0) | 0.04 | 74.2 (70.3, 77.9) | 74.0 (70.4, 78.1) | -0.01 |
| MRR, beats/min | 17.4 (15.8, 19.1) | 17.6 (16.1, 19.4) | -0.07 | 17.5 (16.0, 19.3) | 17.5 (16.0, 19.3) | 0 |
| Temperature, ℃ | 36.7 (36.5, 37.0) | 36.7 (36.5, 36.9) | 0.17 | 36.7 (36.5, 36.9) | 36.7 (36.5, 36.9) | 0.01 |
| **Comorbidities** | | | | | | |
| Hypertension, n(%) | 2,100.0(57.3) | 1,939.0(55.4) | 0.04 | 1,542.0(56.8) | 1,532.0(56.4) | 0.01 |
| Myocardia Infarct, n(%) | 1,025.0(27.9) | 1,049.0(30.0) | -0.04 | 777.0(28.6) | 782.0 (28.8) | 0 |
| Renal Disease, n(%) | 552.0 (15.0) | 632.0(18.0) | -0.08 | 437.0(16.1) | 447.0 (16.5) | -0.01 |
| Cerebrovascular Disease, n(%) | 416.0 (11.3) | 330.0 (9.4) | 0.06 | 284.0(10.5) | 278.0 (10.2) | 0.01 |
| Diabetes, n(%) | 1,277.0(34.8) | 1,263.0(36.1) | -0.03 | 948.0(34.9) | 945.0 (34.8) | 0 |
| Live Disease , n(%) | 154.0 (4.2) | 129.0 (3.7) | 0.03 | 100.0(3.7) | 103.0 (3.8) | -0.01 |
| Chronic pulmonary Disease, n(%) | 903.0 (24.6) | 648.0(18.5) | 0.15 | 569.0(21.0) | 573.0 (21.1) | 0 |
| Peripheral Vascular Disease, n(%) | 655.0 (17.9) | 555.0 (15.8) | 0.05 | 464.0 (17.1) | 451.0 (16.6) | 0.01 |
| Congestive Heart Failure, n(%) | 1,069.0(29.1) | 900.0(25.7) | 0.08 | 734.0(27.0) | 725.0 (26.7) | 0.01 |
| **Scoring systems** | | | | | | |
| ASP-III | 36.0 (28.0, 48.0) | 34.0 (27.0, 45.0) | 0.11 | 35.0 (27.0, 46.0) | 34.0 (27.0, 46.0) | 0 |
| SOFA | 5.0 (4.0, 7.0) | 5.0 (4.0, 7.0) | -0.09 | 5.0 (4.0, 7.0) | 5.0 (4.0, 7.0) | -0.01 |
| **Laboratory tests** | | | | | | |
| WBC, (K/µL) | 12.0 (9.0, 15.8) | 12.3 (9.2, 15.6) | -0.02 | 12.1 (9.1, 16.0) | 12.3 (9.2, 15.5) | 0.02 |
| Chloride, (mEq/L) | 109.0 (107.0, 112.0) | 108.0 (106.0, 110.0) | 0.23 | 109.0 (106.0, 111.0) | 108.0 (106.0, 111.0) | 0.03 |
| Potassium, (mEq/L) | 4.2 (3.9, 4.5) | 4.4 (4.0, 4.7) | -0.26 | 4.3 (3.9, 4.6) | 4.3 (4.0, 4.6) | -0.03 |
| Sodium, (mEq/L) | 139.0 (137.0, 141.0) | 139.0 (137.0, 140.0) | 0.21 | 139.0 (137.0, 141.0) | 139.0 (137.0, 141.0) | 0.03 |
| **Treatment measures** | | | | | | |
| Mechvent Ventilation, n(%) | 2,229.0(60.8) | 2,350.0(67.1) | -0.13 | 1,736.0(64.0) | 1,750.0(64.5) | -0.01 |
| Antibiotics, n(%) | 3,494.0(95.3) | 3,420.0(97.7) | -0.13 | 2,620.0(96.5) | 2,636.0(97.1) | -0.03 |
| Vasoactive Drug, n(%) | 2,812.0(76.7) | 2,545.0(72.7) | 0.09 | 2,039.0(75.1) | 2,026.0(74.6) | 0.01 |

**Notes** OND: Ondansetron; MHR: Mean heart rate; MBP: Mean arterial blood pressure; MRR: Mean respiratory rate; WBC: White Blood Cell; APSIII: Acute physiology score III; SOFA: Sequential Organ Failure Assessment.
